# The cytotoxic synergy between *Clostridioides difficile* toxin B and proinflammatory cytokines: an unholy alliance favoring the onset of *Clostridioides difficile* infection and relapses

**DOI:** 10.1002/mbo3.1061

**Published:** 2020-07-12

**Authors:** Gabrio Bassotti, Andrea Marchegiani, Pierfrancesco Marconi, Katia Fettucciari

**Affiliations:** ^1^ Gastroenterology & Hepatology Section Department of Medicine University of Perugia Medical School Perugia Italy; ^2^ Gastroenterology & Hepatology Unit Santa Maria della Misericordia Hospital Perugia Italy; ^3^ School of Biosciences and Veterinary Medicine University of Camerino Macerata Italy; ^4^ Department of Experimental Medicine University of Perugia Medical School Perugia Italy

**Keywords:** *Clostridioides difficile* infection, infection relapses, proinflammatory cytokines, toxin B

## Abstract

*Clostridioides difficile* infection (CDI) represents an important health problem worldwide, with significant morbidity and mortality. This infection has also high recurrence rates, whose pathophysiological grounds are still poorly understood. Based on our experiments in vitro with *Clostridioides difficile* toxin B and existing experimental and clinical evidence, we propose that primary CDI and relapses might be favored by a mechanism that involves the enhancement of the toxicity of toxin B by proinflammatory cytokines, tumor necrosis factor alpha, and interferon gamma on the enteric glial cells and their network in an environment characterized by a strong dysmicrobism.

## Graphical abstract

Toxin B is a major virulence factor in *Clostridioides difficile* infection (CDI). The second episode of CDI is categorized as either a relapse or a second new infection (reinfection). We hypothesize that relapses might be due to a mechanism that involves the enhancement of the toxicity of toxin B by proinflammatory cytokines on the enteric glial cell network.

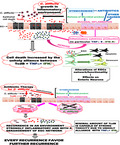

## 1 INTRODUCTION

In the last years, *Clostridioides* (formerly *Clostridium*) *difficile* infection (CDI) has become an important health problem worldwide (Dicks, Mikkelsen, Brandsborg, & Marcotte, 2019; He et al., 2013), causing significant morbidity and mortality in the general population (Lessa et al., 2015). Of note, recurrence rates for this infection average overall 20%, occurring in about 21% of healthcare‐associated infections and about 14% of community‐associated infections (Lessa et al., 2015). The difficulties of treating recurrent infections and the risk of transmission after symptoms' recurrence make these figures worrisome (Belmares, Gerding, Tillotson, & Johnson, 2008; Guery, Galperine, & Barbut, 2019; Singh et al., 2019; Vardakas et al., 2012).

The pathophysiological factors leading to CDI and relapses are still poorly known (Petrosillo, 2018), and it is likely that more than one mechanism may exert some important action (Awad, Johanesen, Carter, Rose, & Lyras, 2014; Gil, Pizarro‐Guajardo, Álvarez, Garavaglia, & Paredes‐Sabja, 2015; Peniche, Savidge, & Dann, 2013). Among these, *Clostridioides difficile* toxins (Tcd, A, and B) play a pivotal role in acting at multiple levels (Aktories, Schwan, & Jank, 2017; Papatheodorou, Barth, Minton, & Aktories, 2018). One of the various cellular targets of the toxins during CDI is represented by the enteric glial cells (EGC), a cell population involved in several important physiologic gut functions, including motility, perception, secretion, neurotransmission, and mucosal permeability (Bassotti, Villanacci, Antonelli, Morelli, & Salerni, 2007a; Bassotti, Villanacci, Fisogni, et al., 2007b; Grubišić & Gulbransen, 2017; Morales‐Soto & Gulbransen, 2019; Vergnolle & Cirillo, 2018).

We have recently demonstrated that in vitro TcdB causes in EGC cytopathic (disruption of cytoskeleton assembly, cell rounding, loss of adhesion, cell cycle arrest) and cytotoxic (apoptosis) effects related to Rac1 glucosylation (Fettucciari et al., 2017); these effects were time‐ and dose‐dependent (Fettucciari et al., 2017). Of interest, EGC that survived the cytotoxic effects of TcdB underwent cellular senescence (Fettucciari et al., 2018) with persistent impaired cell functions.

Most importantly, we showed that the stimulation of EGC with inflammatory cytokines, tumor necrosis factor alpha (TNFα), and interferon gamma (IFNγ) at doses that did not affect control cells, before (−18 hr) or after (+2 hr) TcdB treatment, strongly increased TcdB‐induced apoptosis (Fettucciari et al., 2017). This suggests the inflammatory cytokines could play a key role in vivo in the pathogenesis of CDI (Bassotti, Macchioni, Corazzi, Marconi, & Fettucciari, 2018).

## 2 CYTOTOXIC SYNERGISM BETWEEN TCDB AND TNFα PLUS IFNγ: IN VIVO IMPLICATIONS

Based on our previous in vitro results (Fettucciari et al., 2017) on the enhancement of the toxic action of TcdB on EGCs by the proinflammatory cytokines TNFα and IFNγ independently from the time of cytokine addition (Fettucciari et al., 2017), the possible implications in vivo are thus schematized:

*Experimental situation A*: cytokines present 18 hr before adding TcdB to EGCs.
  In vivo analogy: *C. difficile* colonizes the bowel and then begins infection in individuals with an inflammatory state preceding the infection. Therefore, from the very beginning, the cytotoxic activity of TcdB is enhanced by the proinflammatory cytokines TNFα and IFNγ already present in the gut *milieu*. Conditions that may cause an inflammatory state before CDI are as follows: (a) gastrointestinal tract and respiratory system infections (Svanborg, Godaly, & Hedlund, 1999; Winter, Lopez, & Bäumler, 2013; Wlodarska & Finlay, 2010) and (b) antibiotic therapy which, in addition to causing dysmicrobism, induces the release of structural and metabolic bacterial components able to stimulate the production of cytokines by both the various subtypes of innate immunity and epithelial cells (Ginsburg, 2002; Ginsburg & Koren, 2018; Lepper et al., 2002; Prins, van Deventer, Kuijper, & Speelman, 1994).
*Experimental situation B*: cytokines added 2 hr after EGC intoxication with TcdB.
  In vivo analogy: Immediately after the start of the infection (with production and release of TcdA and TcdB), *C. difficile* induces a response mainly characterized by induction of inflammatory cytokines including TNFα and IFNγ (Aktories et al., 2017; Carter et al., 2015; Czepiel et al., 2014; Feghaly, Bangar, & Haslam, 2015; Feghaly et al., 2013; Johal, Solomon, Dodson, Borriello, & Mahida, 2004; Sun, Savidge, & Feng, 2010), which enhance the toxic action of TcdB. Subsequently, with the growth of the infection, the increased secretion of toxins accompanies the increase in the inflammatory response and therefore the enhancement of the cytotoxic activity of TcdB. A pivotal contribution to the stimulation of innate immunity, and therefore of the increase in proinflammatory cytokines, is also provided by the release of structural and metabolic components (Darkoh, Plants‐Paris, Bishoff, & DuPont, 2019; Hryckowian, Pruss, & Sonnenburg, 2017; Mori & Takahashi, 2018) by *C. difficile*.
*Implications for CDI relapses:* On the abovegrounds, it might be hypothesized that after recovery from the primary infection, the conditions favoring primary infection persist and are also more exasperated from a dysmicrobism accentuated by anti‐*C. difficile* therapy and by a persistent and stronger inflammatory state. Therefore, if *C. difficile* has not been eradicated by the therapy (Vardakas et al., 2012), or there is reinfection, the whole system enhances the action of TcdB, but in conditions of dysmicrobism and proinflammatory responses much more increased (Vardakas et al., 2012). Thus, recovery from a first relapse might render an individual more susceptible to a second recurrence with increasingly serious consequences (Belmares et al., 2008).

## 3 ROLE OF EGC IN RELAPSES

Based on our results, in which EGCs are susceptible to the action of TcdB (Fettucciari et al., 2017), what role could they play in susceptibility to CDI relapses? When developing CDI, the toxins released by the microorganisms firstly interact with the colonic epithelial cells, causing abnormalities of the epithelial barrier and allowing the deep penetration within the bowel wall (Aktories et al., 2017; Feghaly et al., 2015; Johal et al., 2004; Sun et al., 2010). Thus, TcdB may reach the EGC network and exert the toxic effects that depend on the amount of the toxin itself. Based on our in vitro observations (Bassotti et al., 2018; Fettucciari et al., 2017, 2018), we suggest that high doses of TcdB, like those released during a first infection, kill a certain percentage of EGC through apoptotic and necrotic mechanisms (Fettucciari et al., 2017) and lead to senescence in the surviving EGC (Fettucciari et al., 2018) with important alterations of their physiological function, contributing to the acute effects of CDI. However, although in the absence of proinflammatory cytokines, low doses of TcdB had a weak apoptotic effect on EGC (Fettucciari et al., 2017), in the presence of proinflammatory cytokines (TNFα and IFNγ) even low doses of TcdB increase cytotoxic effects (Fettucciari et al., 2017), highlighting the fact that that these cytokines exacerbate the toxicity of TcdB.

The hypothesized main consequences of TcdB action on EGC are the derangement of the EGC network with impairment of the secretory/sensory/motor functions of the large bowel (Bassotti et al., 2018). Besides, EGC surviving the acute effects of TcdB may become senescent (Fettucciari et al., 2018), with loss of functionality, activation of cytokine release, and inhibition of neogliogenesis. Further, the persistence of proinflammatory cytokines favors the persistence of senescent cells (Frey, Venturelli, Zender, & Bitzer, 2018; Hernandez‐Segura, Nehme, & Demaria, 2018; Penfield, Anderson, Lutzke, & Wang, 2013). These changes, in turn, affect other cells (neurons, ICC) that are functionally strictly related to ECG (Bassotti, Villanacci, Antonelli, et al., 2007a; Bassotti, Villanacci, Fisogni, et al., 2007b; Grubišić & Gulbransen, 2017), and weaken the control of the microbiome, worsening the picture and open the way to CDI relapses, either because of the failed eradication of *C. difficile* with antibiotic therapy or because of reinfection. The latter is particularly frequent in old and frail subjects (Meltzer et al., 2019), in whom even minor dysfunctions of intestinal physiology may take up clinical relevance.

## 4 CONCLUSIONS

We hypothesize that the unholy alliance between proinflammatory cytokines and TcdB, consisting of an important synergic toxic effect and abnormalities of functions in surviving EGC, might be one of the pivotal factors of CDI and relapses. We propose that primary CDI and relapses are due to a cytotoxic synergy between TcdB and proinflammatory cytokines (such as TNFα and IFNγ), that develops according to a looping sequence:

1. primary CDI in a subject with a preceding inflammatory microenvironment that boosts the toxicity of TcdB;
2. CDI, which increase the production of proinflammatory cytokines with exacerbation of the effects of TcdB;
3. antibiotic treatment for CDI, with further alteration of the microbiome and increased cytokine production;
4. *C. difficile* residual population not eradicated by therapy or reinfection with an enhancement of pathogenicity due to the persistent presence of proinflammatory cytokines and impaired functionality of the enteroglial network;
5. system increasingly receptive and susceptible to any new recurrence of infection due to a progressive amplification of all the events described above.

We feel that the paramount importance of inflammatory response in CDI and disruption of EGC key cells in regulating directly or indirectly multiple intestinal functions extremely important for the homeostasis of the gut (Bassotti, Villanacci, Antonelli, et al., 2007a; Bassotti, Villanacci, Fisogni, et al., 2007b; Grubišić & Gulbransen, 2017; Vergnolle & Cirillo, 2018) makes the hypothesis that cytokines can be important drivers of CDI and relapse very appealing and suggestive. In light of this, therapy and relapses prevention should intervene for antagonizing the two key events of the CDI, namely the dysmicrobism and the proinflammatory response: (a) reconstitution of microbial flora, for example, with fecal transplantation (Rokkas et al., 2019); (b) moderation of the action of proinflammatory cytokines, to break the loop (unholy alliance) leading to CDI and relapses.

## CONFLICT OF INTEREST

None declared.

## AUTHOR CONTRIBUTION

**Gabrio Bassotti:** Conceptualization (lead); Data curation (equal); Methodology (lead); Supervision (equal); Validation (equal); Writing‐original draft (lead); Writing‐review & editing (equal). **Andrea Marchegiani:** Data curation (supporting); Methodology (supporting); Validation (equal); Writing‐review & editing (supporting). **Pierfrancesco Marconi:** Conceptualization (equal); Data curation (lead); Formal analysis (equal); Methodology (equal); Supervision (lead); Validation (equal); Visualization (equal); Writing‐original draft (equal); Writing‐review & editing (equal). **Katia Fettucciari:** Conceptualization (equal); Data curation (equal); Formal analysis (equal); Methodology (equal); Validation (equal); Writing‐review & editing (equal).

## ETHICS STATEMENT

None required.

## Data Availability

Not applicable.
